# Association between Serum Irisin and Leptin Levels and Risk of Depressive Symptoms in the Diabetic Elderly Population

**DOI:** 10.3390/jcm12134283

**Published:** 2023-06-26

**Authors:** Malgorzata Gorska-Ciebiada, Maciej Ciebiada

**Affiliations:** 1 Department of Propaedeutics of Lifestyle Diseases, Medical University of Lodz, 90-251 Lodz, Poland; 2 Department of General and Oncological Pneumology, Medical University of Lodz, 90-549 Lodz, Poland; maciej.ciebiada@umed.lodz.pl

**Keywords:** irisin levels, leptin levels, elderly, depressive symptoms, type 2 diabetes

## Abstract

Background: Adipokines are considered to be involved in the pathogenesis of diabetes and depression. The associations of serum levels of leptin and irisin with depressive symptoms were investigated in elderly patients with type 2 diabetes (T2DM). Methods: 189 elderly diabetics were assessed with the 30-item Geriatric Depression Scale (GDS-30), and 57 patients with depressive symptoms and 132 controls were selected. Blood biochemical parameters, including serum irisin and leptin, were measured. Results: Serum irisin levels were decreased and leptin concentrations were significantly higher in T2DM patients with depressive symptoms compared to controls. In all subjects, the irisin level was inversely correlated with the leptin level and the GDS-30 score, whereas the leptin level was highly correlated with BMI and the GDS-30 score. Higher levels of leptin and lower concentrations of irisin are, among other factors, variables indicative of predictive capacity for depressive symptoms in elderly patients with T2DM. Conclusions: The results indicated that irisin and leptin levels may be used as diagnostic markers of depressive symptoms in diabetic, elderly patients and as potential therapeutic targets for the treatment. Further prospective and more extensive studies are needed to clarify the role of these adipokines in the common pathogenesis of depression and diabetes.

## 1. Introduction

Both diabetes and depression are major medical concerns generating significant socioeconomic costs. The number of adults who suffer from diabetes has increased dramatically over the last few years, and it is projected to rise from 537 million in 2021 worldwide to 783 million by 2045 [1]. The World Health Organization (WHO) reported that about 350 million people in the world have depression [2]. Diabetes increases the likelihood of developing depression by up to 34%, while depression increases the risk of diabetes by 60% [3,4]. Depression is closely associated with higher medical morbidity and mortality in diabetic patients [5]. Many factors have been implicated in the development of depression and diabetes and they are considered relevant to the bidirectional association of these conditions. Some researchers have suggested that obesity, inappropriate diet, or lack of physical activity are common risk factors for both depression and type 2 diabetes (T2DM) [6,7]. It has been hypothesized that physiological stress associated with diabetes leads to dysregulation of diabetes and the development of depressive symptoms. It is proposed that several mechanisms underlie the relationship between chronic stress, depression, and diabetes [8]. One is hormonal imbalance, including increased cortisol levels and disrupted insulin and GLP-1 signaling due to the activation of the hypothalamic–pituitary–adrenal axis (HPA) [8]. Chronic stimulation of HPA, which takes place in both diabetes and depression, as well as in obesity, is associated with an alteration in monoaminergic pathways and impaired neurotransmitter function (serotonin, dopamine, and noradrenalin) [7,9]. Neuroinflammation is another important factor underlying both diabetes and depression. Chronic hyperglycemia through disturbing the brain–blood barrier in the hippocampus could impair neuroplasticity, induce oxidative stress, and harm neurogenesis [10,11]. It has been hypothesized that impaired mitochondrial function driven by chronic inflammation in diabetes decreases hippocampal neurogenesis, which is crucial in depression [12]. Hippocampal neurogenesis could be influenced by many triggers, such as aging and stress, while it may be increased by physical exercise or antidepressant treatment [13,14]. Irisin, being a novel myokine synthesized in skeletal muscle after exercise, is responsible for neuroplasticity and regulates neuronal differentiation and maturation [15,16]. Overexpression of irisin and its precursor—the fibronectin type III domain-containing protein 5 (FNDC5)—in the hippocampus in mice is induced by physical exercise and has some beneficial anti-depressant, pro-neurogenic, and neuroprotective effects [17]. In addition, a meta-analysis showed that exercise could be a treatment for depression [18]. However, there are hardly any studies including the levels of irisin in patients with T2DM in such a model. Moreover, irisin as the cleaved part of FNDC5 can induce the expression of brain-derived neurotrophic factor (BDNF), which presents neuroprotective effects in depression [19]. Gathered evidence has shown that irisin influences the modulation of glucose and lipid metabolism, and it can also be considered as not only a myokine but also as an adipokine [20]. Numerous studies have shown that it improves sensitivity to insulin by reducing gluconeogenesis and increasing glycogenesis, inhibiting lipogenesis and cholesterol synthesis, and decreasing lipid accumulation and steatosis [21,22,23]. A lot of studies have suggested that adipokines are associated with depression and T2DM [6,7,24]. Low-grade inflammation induced in adipose tissue with increased secretion of cytokines and adipokines is a common factor underlying both obesity, diabetes, and depression [7]. Leptin receptors are widely spread in the brain, mainly in the hypothalamus. Leptin might have a beneficial effect on the nervous system through the modulation of the function of the HPA, the number of neurotransmitters, or neurotrophic factors. On the other hand, obesity and diabetes lead to leptin resistance and leptin signaling disruption. These disturbances are connected with defects in leptin transport through the blood–brain barrier, deficiency of leptin receptors, and dysfunction of the intracellular leptin signaling pathway [24]. 

Some studies suggest that irisin and leptin can play a role in the pathogenesis of concomitant depression in the elderly population. However, data on this issue are limited. In the present study, we investigated the levels of serum irisin and leptin in elderly diabetic patients with and without depressive symptoms. We explored the association between those parameters and other indicators of depressive symptoms based on the findings from the literature. We hypothesized that irisin and leptin concentrations could be, among other factors, variables indicative of predictive capacity for depressive symptoms in elderly patients with T2DM. 

## 2. Materials and Methods

### 2.1. Design

According to the study that was previously described, a total of 189 participants were identified [25]. 

A cross-sectional study was conducted among individuals taken from the diabetes outpatient clinic that is a part of the University Teaching Hospital No 1 in Lodz, Poland. After signing a written informed consent, all subjects underwent a stepwise diagnostic procedure. In the first step, the subjects underwent a complete physical examination and had their height and weight measured. Venous blood samples were drawn in the morning from the fasting participants. Afterwards, the subjects consumed a snack followed by a measurement of capillary glucose level to confirm that patients were not hypoglycemic upon the psychological examination. In the next step, the participants were evaluated in a private area in the clinic. They filled out questionnaires assessing depressive symptoms, underwent a standardized interview, and gave answers regarding baseline demographics.

### 2.2. Study Population

Participants were recruited from the elderly population, aged 65 and above. All subjects had been diagnosed with type 2 diabetes for at least one year, and were capable of understanding and cooperating with study procedures. The exclusion criteria were as follows: previously diagnosed depression or dementia therapy with possible or known cognition-impairing drugs in the previous 3 months, recognized cancer disease, constant alcohol or substance consumption, severe visual, mobility, or motor coordination problems, history of head trauma, inflammatory or infectious brain illness, a severe neurological or psychiatric dysfunction. 

### 2.3. Measurements

Depressive symptoms were recognized using the long version (30-item) of the Geriatric Depression Scale (GDS) [26]. This screening tool is used to evaluate the severity of depression symptoms in older populations. The scale has been appropriately validated to be used in Poland. The questionnaire was self-reported by the majority of the patients. A trained investigator evaluated every subject, interviewing when the patients had eye problems or low-literacy and scored all the answers. The mood states were defined as follows: the normal state was diagnosed for GDS score between 0 and 9; depressive symptoms—for GDS score between 10 and 19; severe depression—for GDS scores ≥ 20 and these patients were further evaluated in a psychiatric clinic. Information on the age, gender, marital status, smoking habits, physical activity, T2DM duration, diabetic complications, co-morbidities, and the treatment was gathered during the medical interview and also collected from the individual’s medical record. Weight and height were measured and Body Mass Index (BMI) was calculated as weight/height^2^ (kg/m^2^). Diabetic vascular complications included the presence of neuropathy, nephropathy, retinopathy, previous cardiovascular disease (CVD), and stroke. Hypertension was defined as either a history of this disease or therapy with any antihypertensive agents. Taking any lipid-lowering agent or an untreated serum LDL cholesterol level of 2.6 mmol/L or/and triglycerides of 1.7 mmol/L were identified as hyperlipidemia. To measure the serum levels of irisin and leptin, fasting blood samples were collected, and stored at a temperature −80 °C until assayed. The irisin concentration was evaluated using an enzyme-linked immunosorbent assay kit (the ELISA kit (Phoenix Pharmaceuticals Inc., Burlingame, CA, USA) and leptin was determined by the Quantikine Human Immunoassay ELISA kit (R & D System, Minneapolis, MN, USA) according to the manufacturer’s protocols. Minimum detectable concentrations were: 1.2 ng/mL for irisin and 7.8 pg/mL for leptin. Other parameters, including total cholesterol, LDL, high-density lipoprotein (HDL), triglycerides, and glycosylated hemoglobin (HbA1c) levels, were determined in a centralized laboratory. A total of 189 T2DM elderly subjects were divided into two groups: patients with depressive symptoms and individuals without depressive symptoms (controls).

### 2.4. Ethics

The study was performed under the guidelines of the World Medical Association’s Declaration of Helsinki. The investigators fully explained all study procedures, and the purpose, nature, and potential risks of the experiments. All subjects were assigned a number for identification to protect their privacy. Prior to enrollment in the study, each subject gave written, informed consent. The study was approved by independent local ethics committee of the Medical University of Lodz (No. RNN/420/13/KB). 

### 2.5. Statistical Analysis

The Shapiro–Wilk test was used to evaluate the normality of the sample distribution. Categorical variables were extracted as numbers and percentages, whereas continuous variables were as means with standard deviations (SD). The unpaired *t*-tests and the Mann–Whitney U tests were conducted for the parametric and nonparametric continuous variables, respectively. The χ^2^ test was performed for the categorical variables. The Pearson correlation analysis for normally distributed parameters and the Spearman rank correlation for nonnormally distributed parameters were conducted to assess relationships between levels of markers and other variables. The simple logistic regression model was applied in order to select so-called independent factors that increase the selection risk of depressive symptoms in elderly patients with T2DM. Odds ratios (OR) were computed and presented with the 95% interval of confidence (CI). *p* value < 0.05 was adopted as statistically significant. The data were analyzed by the Statistica 13.1 software (StatSoft, Kraków, Poland). 

## 3. Results 

### 3.1. General Demographic, Clinical, and Metabolic Parameters

Table 1 summarizes the demographic, clinical, and metabolic parameters of 189 elderly patients with T2DM in this study. Subjects with depressive symptoms were more likely to be single female smokers. Moreover, they were less physically active and demonstrated higher BMI levels. No significant difference was observed across ages. Furthermore, the *t*-test or Mann–Whitney U test revealed that depressive patients had a longer duration of diabetes, a higher number of chronic diseases, were more often diagnosed with neuropathy and hyperlipidemia, and were treated with insulin rather than administered oral anti-diabetic drugs. The GDS-30 score was significantly higher in subjects with depressive symptoms in comparison with controls. The level of total cholesterol and LDL cholesterol was higher in the group with depressive symptoms. Lastly, no significant differences were found between the groups regarding concentrations of HbA1c, triglycerides, and HDL cholesterol.

### 3.2. Irisin and Leptin Levels in Depressive Patients and Controls

**Figure 1:**
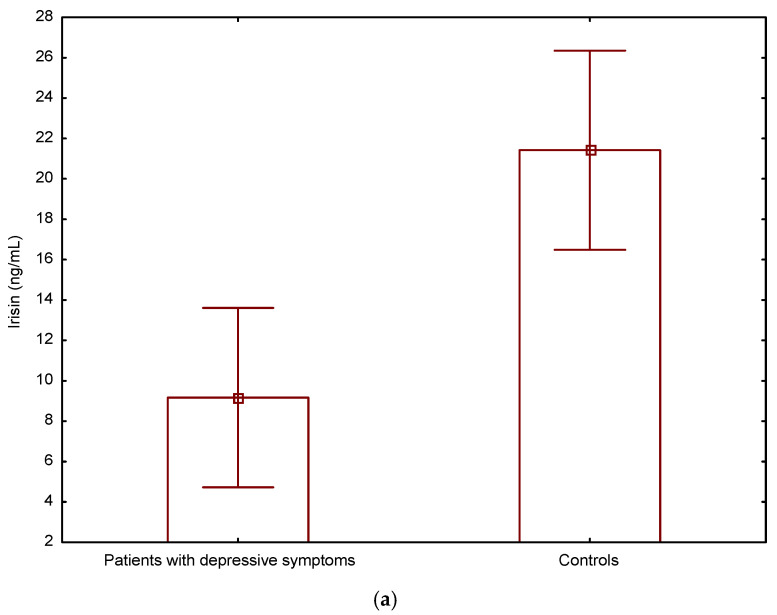

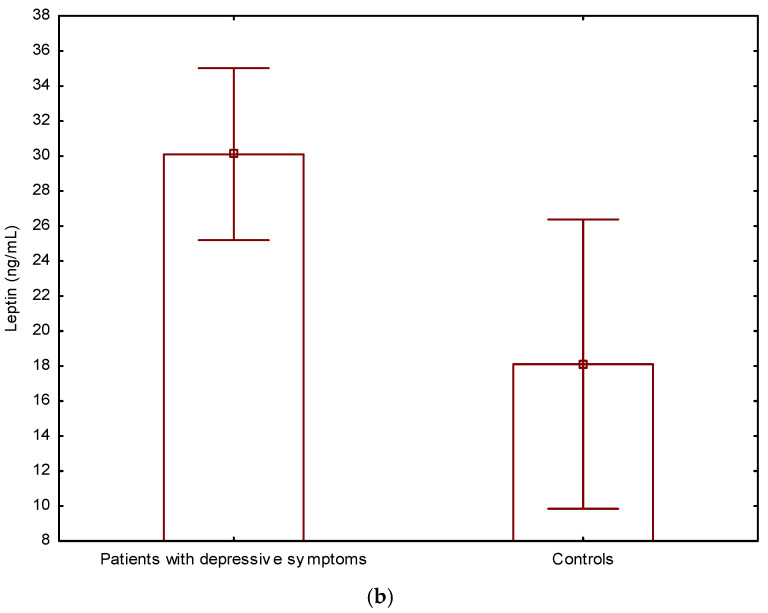
Serum levels of Irisin (ng/mL) (**a**) and Leptin (ng/mL) (**b**) in T2DM elderly patients with and without depressive symptoms.

The serum irisin level was significantly decreased in patients with depressive symptoms compared to controls (*p* < 0.001) (Figure 1a). The mean level of irisin in the depressive group was 9.17 ± 4.4 ng/mL compared to the level of 21.4 ± 4.9 ng/mL in controls (*p* < 0.001); all subjects—17.7 ± 7.4 ng/mL. 

A correlation analysis showed a significant negative relationship between irisin levels and total cholesterol and LDL cholesterol in all studied groups (Table 2). In all subjects, the irisin concentration was highly inversely correlated with the GDS-30 score. Furthermore, we noticed a weak inverse correlation between irisin levels and BMI (in all subjects), number of co-morbidities (in all subjects and in depressive patients), age (in depressive subjects), triglycerides (in depressive subjects), and duration of diabetes (in all subjects and in the control group) (Table 2). 

Serum leptin level was significantly increased in patients with depressive symptoms compared to controls (*p* < 0.001) (Figure 1b). The mean level of leptin in the depressive group was 30.1 ± 4.9 ng/mL in comparison to the level of 18.1 ± 8.3 ng/mL in controls (*p* < 0.001); all subjects—21.7 ± 9.2 ng/mL (Table 2). In all subjects and both studied groups, the leptin concentration was highly correlated with BMI. We also noticed a significant positive relationship between leptin levels and the GDS-30 score in all subjects and in depressive patients. Furthermore, we showed a weak correlation between leptin levels and the number of co-morbidities (in all subjects), age (in all subjects and in controls), LDL cholesterol (in all subjects and in depressive patients), and duration of diabetes (in all subjects). 

**Figure 2:**
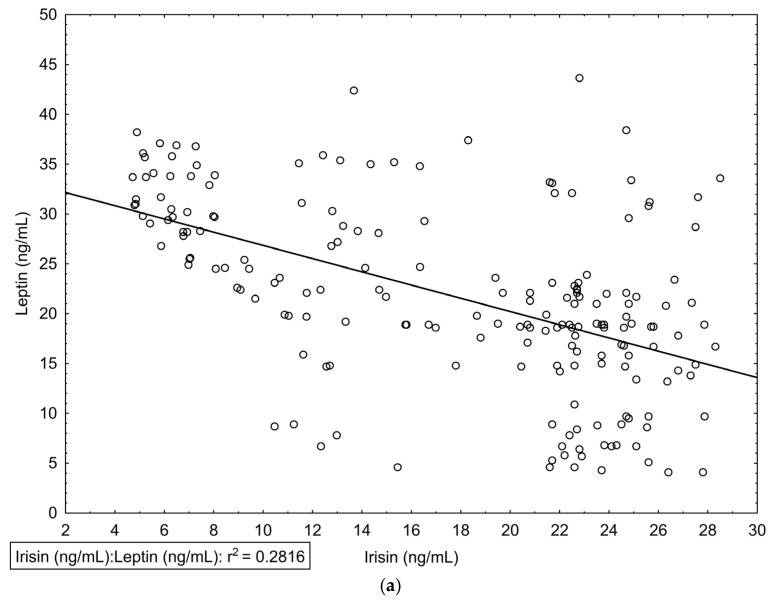

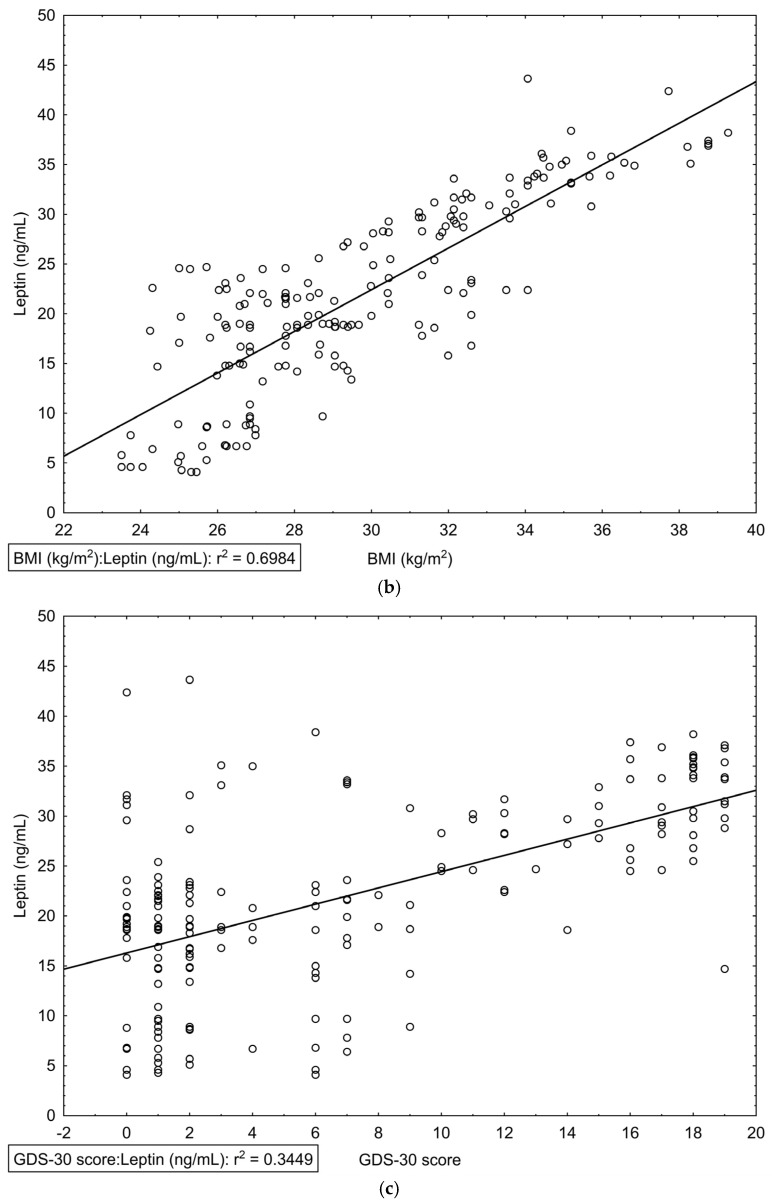

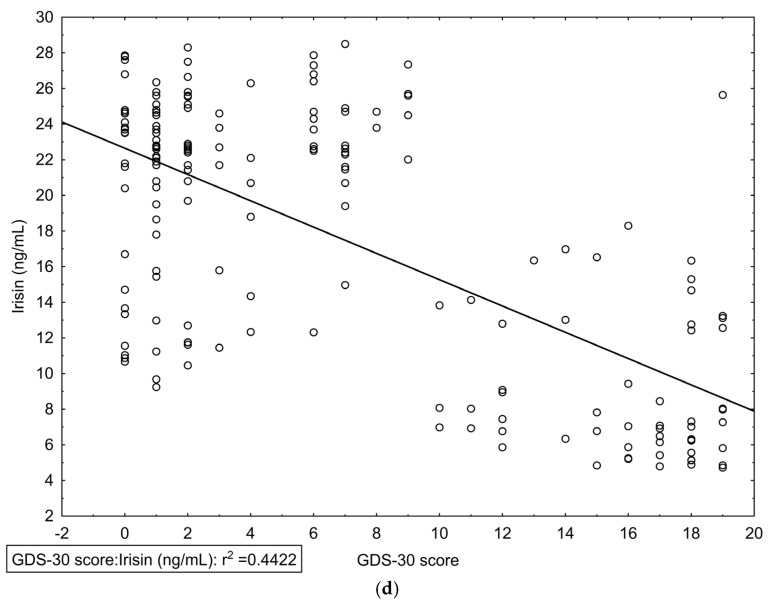
Baseline correlations between (**a**) serum levels of Leptin (ng/mL) and irisin concentrations (ng/mL), (**b**) Leptin (ng/mL) and BMI (kg/m^2^), (**c**) Leptin (ng/mL) and GDS-30 score, and (**d**) irisin concentrations (ng/mL) and GDS-30 score.

Lastly, we found that the serum irisin level is negatively correlated with the leptin level in all subjects. Results are presented in Table 2, and the most statistically significant correlations are presented in Figure 2.

### 3.3. Logistic Regression Models

The univariate logistic regression models showed that variables indicative of predictive capacity for depressive symptoms in elderly patients with type 2 diabetes were female gender, single marital status, smoking, lack of physical activity, higher BMI, longer duration of diabetes, higher number of co-morbidities, diagnosis of neuropathy and hyperlipidemia, insulin treatment, higher levels of leptin, total cholesterol and LDL, and lower concentrations of irisin (Table 3).

## 4. Discussion

Depression and diabetes are among the most prevalent chronic illnesses worldwide [27]. Diabetic patients have depression two to three times more frequently compared to healthy subjects [27]. The presence of depression increases the risk of microvascular and macrovascular complications, generates medical costs, worsens metabolic control, reduces the patient’s quality of life, and is associated with an increased risk of all-cause mortality [28,29]. In recent years, many studies have focused on risk factors of depression in patients with diabetes and potential pathophysiological mechanisms, accelerating the development of diabetes and depression. Besides many hypotheses, the background of the coexistence of these diseases and their common pathogenesis is still unclear. 

In our study, we found that patients with depressive symptoms were mostly single smoking females who were less physically active and demonstrated higher BMI levels. These risk factors of depression in diabetes are commonly described in many previous studies [30,31]. Women are more likely to be sensitive and are prone to depression and anxiety. Living alone, which is more common in elderly populations, promotes loneliness and depressed mood. In the present study, the univariate logistic regression showed that the variables indicative of predictive capacity for depressive symptoms in elderly patients with T2DM were longer duration of diabetes, higher number of co-morbidities, and diagnosis of neuropathy and hyperlipidemia. Complications of diabetes and co-morbidities are other predictors of depressive symptoms in diabetic patients. These can cause a feeling of worthlessness and despair, inferiority, and can affect the self-care ability of elderly patients. The greater number and severity of complications and comorbid diseases, as well as a more difficult and required schema of treatment, e.g., insulin therapy, may cause a great physiological and economic burden on patients, resulting in worsened quality of life.

In the present study, all subjects had an increased BMI, particularly those from the depressive group. Patients with depressive symptoms also performed physical activity less often than controls. Obesity is also an important and well-known risk factor for depression. Patients with diabetes demonstrate a higher prevalence of obesity. A systemic meta-analysis review of longitudinal studies showed that obesity may correlate with depression [32]. Recently published studies have revealed that depression is more frequent in obese elderly diabetics than in normal-weight subjects (49.1% vs. 20.47%) [33]. The authors hypothesized that the relationship between depression, obesity, and physical activity in the elderly population with T2DM is driven through inflammation. 

Recently published studies have paid attention to the increasing role of irisin in the pathophysiology of depression [34,35,36]. Irisin, being an exercise-induced hormone, involved in many metabolic pathways, plays a role in modulating the function of adipose tissue [15]. This myokine induces the process of browning white adipose tissue and stimulates thermogenesis. The role of irisin is also to increase energy expenditure through the upregulation of the expression of PPARα and uncoupling protein-1 in white adipose tissue [22,23]. Irisin can also stimulate in skeletal muscle cells the membrane translocation of GLUT4 (glucose transporter type 4) and induce glucose uptake via stimulating AMP-activated protein kinase phosphorylation [23,37]. Thus, irisin mediates some beneficial effects of exercise. The molecule improves hepatic glucose and lipid metabolism, promotes beta-cell proliferation, and influences beta-cell function [38]. Irisin is mostly derived from muscle in a healthy state. However, in obesity, activated white adipose tissue becomes the second main source of irisin [39]. Irisin concentrations were found to be elevated in subjects with obesity and positively correlated with waist circumference, BMI, waist-to-hip ratio, and fat mass [40,41]. This higher level of adipomyokines in dysmetabolic impairment, such as obesity, may be possibly explained by the state of irisin resistance, similar to insulin and leptin resistance observed in T2DM. The fact that irisin acts as a counterbalancing mechanism by increasing energy expenditure and improving insulin sensitivity might be another explanation. The authors suggested that irisin levels correspond to the amount of white adipose tissue, apart from physical activity and energy loss [40].

We showed that the serum irisin levels were significantly decreased in patients with depressive symptoms compared to controls. There is no other research that describes this marker in diabetic populations with depression. The majority of the studies revealed that patients with type 2 diabetes demonstrate decreased irisin levels [41,42,43,44]. However, some studies performed on obese subjects with T2DM have reported elevated concentrations of this hormone [45,46]. One study showed that decreased concentrations of irisin in individuals with newly diagnosed diabetes correlated with BMI, HbA1c, and triglyceride levels. Moreover, its authors also found significant inverse associations between irisin concentrations and T2DM development [47]. Irisin levels are also associated with diabetic complications. Liu et al. presented significantly lower concentrations of irisin in individuals with diabetes and renal dysfunction [48]. Another study showed lower serum and vitreous irisin levels in patients with proliferative diabetic retinopathy compared to controls [49]. 

The potential role of irisin in the central nervous system is still under investigation. The fact that irisin induces the expression of neurotrophic factors, such as brain-derived neurotrophic factor (BDNF) may be a possible explanation. BDNF can modulate the transcription and transport of mRNA along dendrites, and it can influence the differentiation, growth, and survival of neurons [50,51]. Thus, irisin can promote neuroprotective effects on neurodegenerative diseases such as depression related to neurogenesis [16]. An in vitro study showed that irisin administrated at pharmacological doses elevates the proliferation of neuronal cells in the mouse hippocampus [52]. Irisin also exhibits beneficial protective actions in synapses and memory in Alzheimer’s disease models [20]. 

Interplay with inflammation markers is another mechanism by which irisin mediates depressive mood. In the mouse model, irisin inhibits the secretion of tumor necrosis factor α (TNF-α) and interleukin 6 (IL-6) and reduces neuronal damage induced by oxidative stress [53]. Inflammation can be a common pathomechanism for diabetes, depression, and obesity. Depressive symptoms in diabetes can result from the interplay of other adipomyokines such as leptin, adiponectin, or myoststin.

A recently published study showed that in vitro irisin decreased the expression and production of leptin, adiponectin, TNF-α, IL-6, and macrophage chemotactic protein (MCP-1) [54]. 

Leptin is an adipocyte-derived hormone that is responsible for feelings of satiety. In obesity, the leptin level is pathologically increased due to the desensitization of leptin receptors. The consequence of this process is a depletion of negative feedback on both satiety signaling and leptin production [55]. A state of leptin resistance also is observed in patients with major depression disorder [56]. However, epidemiological studies investigating leptin concentrations in depression are inconsistent, as they can be either lower or higher levels [57,58]. Moreover, circulating levels of leptin also depend on factors such as age, gender, medication history, and food intake [59]. Many experimental studies indicate that leptin could be a modulator of many neurological diseases, including neurodegenerative diseases and mood disorders [24]. Leptin might influence levels of neurotransmitters via the GABAergic system and may influence motivated behavior and reward-seeking behavior through the dopamine pathway [24]. Moreover, leptin might increase the expression of BDNF mRNA and activate BDNF-expressing hypothalamic neurons, thereby influencing hippocampal synaptic plasticity [59]. Lastly, leptin might reverse the impairment of the HPA, which is abnormally activated in depression [59]. Although results of in vitro and in vivo studies revealed that leptin might have a potential anti-depressant function, its beneficial role could be diminished in long-lasting metabolic disorders leading to leptin signaling disruption and leptin resistance [60]. Many studies have indicated that leptin resistance could be the effect of increased endoplasmic reticulum stress in the hypothalamus with leptin signaling dysfunction, defects in leptin passage via the blood–brain barrier, or intracellular leptin pathway disruption [59]. In this study, we showed that serum the leptin level was significantly elevated in individuals with depressive symptoms compared to controls. We also noticed a significant positive relationship between leptin concentrations and the GDS-30 score. These results are consistent with a previous study that revealed a positive association between depression scores and serum leptin levels in patients with T2DM along with leptin resistance [61]. We also found that leptin levels are highly correlated with BMI. Obesity is linked to higher leptin concentrations and the presence of the state of leptin resistance. On the other hand, depression is also associated with obesity. Thus, it seems that the relationship between depression, diabetes, and obesity is complex and could partly be explained by leptin resistance. 

According to another hypothesis, elevated levels of leptin could act as an immunomodulating agent that modulates immune response. Leptin increases the proliferation and activation of macrophages and, thus, can indirectly promote the production of proinflammatory mediators [62]. Proinflammatory mediators are also elevated in individuals with depression and they are also one of the main triggers involved in the pathogenesis of obesity and diabetes [8]. Low-grade systemic inflammation observed in depression, obesity, and diabetes could be driven by increased leptin levels, as well as decreased irisin concentrations, followed by loss of anti-inflammatory properties. 

Higher levels of leptin in depressed individuals could be also explained by lower levels of irisin. We found that the serum irisin level is negatively correlated with leptin level, and as was mentioned before, irisin might inhibit the expression and release of leptin [54]. Finally, we determined the potential of irisin and leptin levels as diagnostic tools for the risk of depressive symptoms in T2DM elderly patients in logistic regression analyses. 

This study has several limitations. Firstly, as our population had depressive symptoms according to the GDS-30 score, patients with severe depression were not subject to evaluation. Thus, further investigations are needed to determine levels of irisin and leptin in those individuals. 

Secondly, this study was a sub-analysis using the subjects from another study. Thus, as the sample consists only of elderly diabetics, the findings are not applicable to the general population as a whole. There is only a univariate analysis and no multivariate analysis adjusted for confounding factors; therefore, some results may be false positives due to type 1 errors. 

Thirdly, we did not evaluate leptin resistance in our study but this could have helped to better understand to what extent elevated leptin levels and leptin resistance contribute to depression in diabetic patients. Assessment of the HPA axis, BDNF, or inflammatory markers could have also helped to clarify the pathophysiology of mood disorders.

Furthermore, the quality of commercially available ELISA kits for circulating irisin was skepticized. Moreover, it was suggested that the validation of the irisin values toward tandem mass spectrometry was a better option to validate the irisin values toward tandem mass spectrometry [63]. 

## 5. Conclusions

In conclusion, we found that serum irisin levels were decreased and leptin concentrations were elevated in elderly diabetic patients with depressive symptoms compared to controls. The variables indicative of predictive capacity for depressive symptoms in elderly patients with T2DM are female gender, single marital status, smoking habit, lack of physical activity, higher BMI, longer duration of diabetes, higher number of co-morbidities, presence of neuropathy and hyperlipidemia, insulin treatment, higher levels of leptin, total cholesterol and LDL, and lower concentrations of irisin.

Our findings suggest that irisin and leptin interplay in regulating mood in elderly patients with diabetes, in addition to their role in metabolic homeostasis, may be used as a diagnostic biomarker of depressive symptoms and serve as a potential therapeutic target. A longitudinal, prospective, normal controlled study evaluating the association between these adipokines and the severity of depression in diabetic individuals is needed to discover the underlying mechanism. 

## Figures and Tables

**Table 1:** Demographic, clinical, and metabolic parameters of T2DM elderly patients.

Parameter	All Subjects (*n* = 189)	Patients with Depressive Symptoms (*n* = 57)	Controls (*n* = 132)	*t*/Z/χ^2^	*p*
Gender (M/F) *	61/128	11/46	50/82	29.2	<0.001
Age (years)	72.6 ± 4.7	72.9 ± 4.0	72.5 ± 4.9	−0.27	0.78
Had ever smoked (%) *	69 (36.5%)	33 (57.8%)	34 (25.7%)	17.9	<0.001
Single/married *	82 (43.4%)	37/20	45/87	15.4	<0.001
No physical activity (%) *	79 (41.8%)	43 (75.43%)	36 (27.3%)	37.9	<0.001
T2DM duration (years) *	7.51 ± 4.7	9.96 ± 6.8	6.45 ± 5.07	3.92	<0.001
Neuropathy (%) *	36 (19%)	23 (40.35%)	13 (9.8%)	24.0	<0.001
Nephropathy (%)	54 (28.6%)	16 (28.07%)	38 (28.8%)	0.01	0.92
Retinopathy (%)	60 (31.7%)	17 (29.8%)	43 (32.6%)	0.14	0.71
Previous CVD (%)	38 (20.1%)	10 (17.54%)	28 (21.2%)	0.33	0.56
Stroke (%)	7 (3.7%)	2 (3.5%)	5 (3.78%)	0.01	0.93
Hyperlipidemia (%) *	138 (73%)	52 (91.22%)	86 (65.15%)	13.7	<0.001
Hypertension (%)	132 (69.8%)	39 (68.42%)	93 (70.4%)	0.08	0.78
Co-morbidity (*n*) *	3.6 ± 2.3	5.3 ± 2.5	2.8 ± 1.8	7.74	<0.001
OAD (%) *	172 (91%)	46 (80.7%)	126 (95.4%)	65.9	<0.001
Insulin (%) *	88 (46.5%)	41 (71.9%)	47 (35.6%)	21.1	<0.001
HbA1c (%)	7.02 ± 0.5	7.06 ± 0.6	7 ± 0.5	0.67	0.49
Total cholesterol (mmol/L) *	4.79 ± 1.0	5.57 ± 0.9	4.46 ± 0.8	7.97	<0.001
Triglycerides (mmol/L)	1.87 ± 0.4	1.86 ± 0.4	1.88 ± 0.3	0.01	1.01
LDL (mmol/L) *	2.83 ± 0.8	3.3 ± 0.8	2.63 ± 0.7	5.75	<0.001
HDL (mmol/L)	1.24 ± 0.2	1.25 ± 0.2	1.24 ± 0.2	−0.74	0.45
BMI (kg/m^2^) *	29.6 ± 3.7	32.1 ± 3.7	28.6 ± 3.1	6.62	<0.001
GDS-30 score	6.7 ± 6.6	15.9 ± 2.8	2.7 ± 2.6	30.9	<0.001
Irisin (ng/mL)	17.7 ± 7.4	9.17 ± 4.4	21.4 ± 4.9	−16.1	<0.001
Leptin (ng/mL)	21.7 ± 9.2	30.1 ± 4.9	18.1 ± 8.3	10.2	<0.001

* Statistically significant difference, *p* < 0.05; comparing individuals with depressive symptoms and controls. Data are presented as mean ± SD values. *t*-test (*t*), Mann–Whitney U test (Z), or χ^2^ test were used to determine significant differences. Abbreviations: T2DM—diabetes type 2; M—male; F—female; CVD—cardiovascular disease; OAD—oral antidiabetic drugs; HbA1c—glycosylated hemoglobin; LDL—low-density lipoprotein; HDL—high-density lipoprotein; BMI—body mass index; GDS—Geriatric Depression Scale.

**Table 2:** Analysis of the correlation between irisin and leptin and other quantitative variables in T2DM elderly patients.

	All Subjects (*n* = 189)				Patients with Depressive Symptoms (*n* = 57)				Controls (*n* = 132)			
Parameter	Irisin r	*p*	Leptin r	*p*	Irisin r	*p*	Leptin r	*p*	Irisin r	*p*	Leptin r	*p*
Age (years)	−0.06	0.38	0.15 *	0.03	−0.3 *	0.02	−0.05	0.69	0.02	0.79	0.21 *	0.02
T2DM duration (years)	−0.32 *	<0.001	0.22 *	0.002	−0.06	0.62	0.01	0.95	−0.23 *	0.008	0.1	0.24
Co-morbidity (*n*)	−0.40 *	<0.001	0.34 *	<0.001	−0.27 *	0.04	0.07	0.57	0.06	0.46	0.07	0.42
HbA1c (%)	−0.08	0.26	0.03	0.66	−0.2	0.134	0.18	0.16	−0.007	0.93	0.05	0.53
Total cholesterol (mmol/L)	−0.78 *	<0.001	0.36 *	<0.001	−0.64 *	<0.001	0.21	0.12	−0.77 *	<0.001	0.05	0.56
Triglycerides (mmol/L)	−0.04	0.58	0.001	0.93	−0.35 *	0.007	0.03	0.85	0.02	0.74	0.01	0.9
LDL (mmol/L)	−0.63 *	<0.001	0.27 *	<0.001	−0.44 *	0.001	0.27 *	0.04	−0.62 *	<0.001	0.001	0.93
HDL (mmol/L)	−0.04	0.57	−0.16	0.25	0.02	0.89	−0.03	0.78	−0.04	0.59	−0.26	0.25
BMI (kg/m^2^)	−0.4 *	<0.001	0.83 *	<0.001	−0.20	0.12	0.94 *	<0.001	−0.08	0.36	0.80 *	<0.001
Irisin (ng/mL)	1		−0.53 *	<0.001	1		0.19	0.14	1		−0.13	0.12
Leptin (ng/mL)	−0.53 *	<0.001	1		0.19	0.14	1		−0.13	0.12	1	
GDS-30 score	−0.67 *	<0.001	0.58 *	<0.001	−0.04	0.78	0.42 *	0.01	−0.19 *	0.02	0.05	0.58

* Statistically significant difference, *p* < 0.05; r—correlation coefficient. Abbreviations: T2DM—diabetes type 2; HbA1c—glycosylated hemoglobin; LDL—low-density lipoprotein; HDL—high-density lipoprotein; BMI—body mass index; GDS—Geriatric Depression Scale.

**Table 3:** Univariate logistic regression analysis of variables indicative of predictive capacity for depressive symptoms in T2DM elderly patients.

Parameter	ß	SE of ß	*p* Value	OR	95% CI
Gender (M/F) *	1.9	0.3	*p* < 0.001	6.8	3.3–14.4
Age (years)	0.2	0.03	0.6	1.0	0.9–1.0
Had ever smoked (%) *	1.4	0.3	*p* < 0.001	3.9	2.1–7.6
Single/married *	1.3	0.3	*p* < 0.001	3.6	1.8–6.8
No physical activity (%) *	2.1	0.3	*p* < 0.001	8.1	4.0–16.7
T2DM duration (years) *	0.1	0.03	*p* < 0.001	1.1	1.0–1.2
Neuropathy (%) *	1.8	0.3	*p* < 0.001	6.2	2.8–13.5
Nephropathy (%)	0.3	0.03	0.92	0.9	0.4–1.9
Retinopathy (%)	0.3	0.01	0.71	0.9	0.4–1.7
Previous CVD (%)	0.2	0.4	0.56	0.8	0.4–1.7
Stroke (%)	0.1	0.8	0.93	0.9	0.2–4.9
Hyperlipidemia (%) *	1.7	0.5	*p* < 0.001	5.6	2.1–14.8
Hypertension (%)	0.1	0.3	0.78	0.9	0.4–1.7
Co-morbidity (*n*) *	0.5	0.08	*p* < 0.001	1.7	1.4–1.9
OAD (%) *	−3.2	0.4	*p* < 0.001	0.03	0.01–0.09
Insulin (%) *	1.53	0.3	*p* < 0.001	4.6	2.4–9.1
HbA1c (%)	0.2	0.2	0.5	1.2	0.6–2.1
Total cholesterol (mmol/L) *	0.03	0.001	*p* < 0.001	1.03	1.02–1.04
Triglycerides (mmol/L)	0.01	0.005	0.79	0.99	0.98–1.01
LDL (mmol/L) *	0.02	0.006	*p* < 0.001	1.03	1.01–1.04
HDL (mmol/L)	0.08	0.02	0.69	1.01	0.9–1.1
BMI (kg/m^2^) *	0.3	0.05	*p* < 0.001	1.3	1.2–1.5
Irisin (ng/mL) *	−0.4	0.05	*p* < 0.001	0.69	0.62–0.76
Leptin (ng/mL) *	0.2	0.03	*p* < 0.001	1.2	1.2–1.33

* Statistically significant difference, *p* < 0.05; ß: regression coefficient; SE: standard error; OR: odds ratio; CI: confidence interval for odds ratio; Abbreviations: T2DM—diabetes type 2; M—male; F—female; CVD—cardiovascular disease; OAD—oral anti-diabetic drug; HbA1c—glycosylated hemoglobin; LDL—low-density lipoprotein; HDL—high-density lipoprotein.

## Data Availability

The data presented in this study are available on request from the corresponding author.
